# Water lilies as emerging models for Darwin’s abominable mystery

**DOI:** 10.1038/hortres.2017.51

**Published:** 2017-10-04

**Authors:** Fei Chen, Xing Liu, Cuiwei Yu, Yuchu Chen, Haibao Tang, Liangsheng Zhang

**Affiliations:** 1 Center for Genomics and Biotechnology; State Key Laboratory of Ecological Pest Control for Fujian and Taiwan Crops; Key Laboratory of Ministry of Education for Genetics, Breeding and Multiple Utilization of Crops; Fujian Provincial Key Laboratory of Haixia Applied Plant Systems Biology; Fujian Agriculture and Forestry University, Fuzhou 350002, China; 2 Zhejiang Humanities Landscape Co., LTD, Hangzhou 310030, China

## Abstract

Water lilies are not only highly favored aquatic ornamental plants with cultural and economic importance but they also occupy a critical evolutionary space that is crucial for understanding the origin and early evolutionary trajectory of flowering plants. The birth and rapid radiation of flowering plants has interested many scientists and was considered ‘an abominable mystery’ by Charles Darwin. In searching for the angiosperm evolutionary origin and its underlying mechanisms, the genome of *Amborella* has shed some light on the molecular features of one of the basal angiosperm lineages; however, little is known regarding the genetics and genomics of another basal angiosperm lineage, namely, the water lily. In this study, we reviewed current molecular research and note that water lily research has entered the genomic era. We propose that the genome of the water lily is critical for studying the contentious relationship of basal angiosperms and Darwin’s ‘abominable mystery’. Four pantropical water lilies, especially the recently sequenced *Nymphaea colorata,* have characteristics such as small size, rapid growth rate and numerous seeds and can act as the best model for understanding the origin of angiosperms. The water lily genome is also valuable for revealing the genetics of ornamental traits and will largely accelerate the molecular breeding of water lilies.

## Introduction

### Ornamentals, cultural symbols and economic value

**Figure 1:**
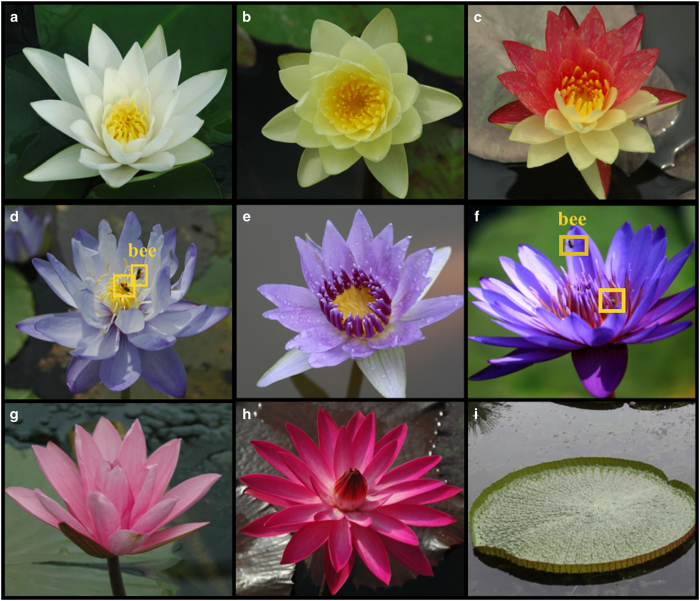
Water lilies are ornamental plants with beautiful flowers and leaves: (**a**) *Nymphaea* ‘Hermine’, (**b**) *N*. ‘Marliacea Chromatella’, (**c**) *N.* ‘Wanvisa’, (**d**) *N.* ‘Gigantea Hybrid1’, (**e**) *N.* colorata, (**f**) *N.* ‘Muang Wiboonlak’, (**g**) *N*. ‘Piyalarp’, (**h**) *N*. ‘Agkee Sri Non’, (**i**) leaf ornamental Victoria water lily.

Water lilies are beautiful aquatic flowering plants that are distributed worldwide. These plants are found in the aquatic section of nearly every botanic garden because of their highly valued ornamental features. The almost full spectrum of petal colors range from black to white, making water lilies the most diversely colored flowering plants (Figure 1). The lovely cup-like flower shapes and floating leaves such as the famous Victoria and Amazon water lilies are also favored ornamental characteristics. In Bangladesh and Sri Lanka, water lilies were chosen as the national flower because they are regarded as a symbol of truth, purity, and discipline.

Beyond being beautiful ornamental plants, water lilies have been utilized as an ingredient in many products, including beneficial and cosmetic substances, soap, perfume, hand cream, flower tea bags, and traditional medicine.^1^ In Asian countries, some water lilies such as *Brasenia schreberi*, *Euryale ferox*, *Nymphaea* spp. are traditional vegetables with edible parts including young leaves, stems, and seeds. Several *Nymphaea* species have also been used to purify heavy metal-contaminated water and soap-polluted wastewater.^2^

### Critical evolutionary place

In taxonomy, plants categorized in the Order Nymphaeales share the common name water lily.^3^ Water lilies are divided into three families: Hydatellaceae, Cabombaceae, and Nymphaeaceae.^4^ The family Nymphaeaceae has the most species of the three families and consists of six genera: *Barclaya*, *Euryale*, *Nuphar*, *Nymphaea*, *Ondinea*, and *Victoria*.^4,5^ Floral organs differ greatly among each family in the order Nymphaeales. In the genus *Nymphaea*, flowers are composed of 4 sepals, 50 to 70 petals, 30 to 40 carpels, and 120 to 250 stamens. These characteristics are often regarded as the most primitive angiosperm floral characteristics, as seen in various ancestral flowering plant fossils.^6^

In the tree of plant life, basal angiosperms consisting of three orders Nymphaeales, Amborellales, and Austrobaileyales, have long been regarded as the basal branches of angiosperms using both molecular phylogenetic and developmental classifications.^7,8^ Although multiple lines of evidence support *Amborella* as the basal-most angiosperm,^7,9–11^ the water lily-basal or *Amrorella*-water lily co-basal theories cannot yet be ruled out.^12–15^ The genomic sequences of the water lily may be critical in resolving the early evolution of angiosperms, because among all basal angiosperms only the genome of *Amborella* is currently known.

### Limited genetic and genomic analysis of water lilies

Despite the importance of water lilies in phylogenetic research and as an aquatic ornamental plant, limited genetic and genomic information is available. Previous chromosome number and size studies have provided the karyotype background of approximately 65 water lily species ^16,17^ (Table 1). Only two homologs of *INO* genes,^18^ two reference genes for expression studies,^19^ six floral organ identity genes,^20^ and ABC model genes ^21^ have been cloned (Table 1). Genetic markers, such as the *matK* genes ^4^ and inter-simple sequence repeats ^22^ have been applied in DNA barcoding of the water lily germplasm.

At the omics level, genome-wide expressed sequence tags (ESTs) were generated in 2006 from the yellow water lily *Nuphar advena* for genome duplication analysis.^23^ Later, the transcriptomes from seven tissues/organs were sequenced and analyzed from the same species^24^ (Table 1). Recently, the transcriptome of six samples from two coloring stages of the beautiful blue water lily *Nymphaea* ‘King of Siam’ were sequenced, together with metabolic analysis, to reveal the blue flower’s formation.^25^ So far, no water lily genome has been reported.

### The water lily holds the key to Darwin’s abominable mystery

The origin and rapid massive expansion of flowering plants in a relatively short geological time, which resulted in most current-day flora, fascinated Charles Darwin, who called it an ‘abominable mystery’ and the ‘most perplexing phenomenon’, beyond which there was ‘nothing... more extraordinary’.^26^ Over the 137 years since this expansion was proposed by Charles Darwin in 1879, evolutionary biologists have long attempted to reconstruct the early history of angiosperms. One of the most critical questions to solving this mystery is to determine which lineage is the most basal angiosperm. So far, there have been several hypotheses.

#### From ANITA to ANA basal hypothesis to Amborella and water lily co-basal hypothesis

**Figure 2:**
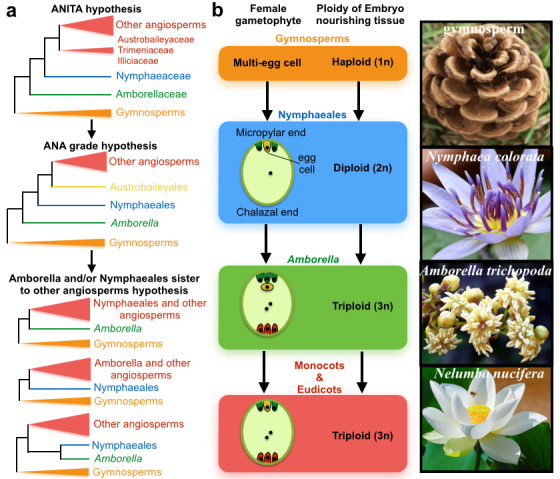
Phylogenetic uncertainty among *Amborella*, water lily, and other angiosperms. (**a**) Hypothesized phylogenetic relationships of basal angiosperms. (**b**) Developmental evidence suggests water lily as the most basal angiosperm.

In 1999, relying on molecular phylogenetic methods, several groups proposed that the *Amborella*, Nymphaeales, and Illiciales-Trimeniaceae-Austrobaileya (ANITA) clade is the extant basal angiosperm^8,27,28^ (Figure 2). However, these phylogenetic trees were all based on a single gene or a few genes, mainly from chloroplasts.^28^ In 2005, based on several plastid, mitochondrial, and nuclear genes, researchers proposed that the *Amborella*, Nymphaeaceae, and Austrobaileyales (ANA) clade (Figure 2) were the basal sister clades to all other angiosperms.^29^ This classification sets either *Amborella* or *Amborella* and Nymphaeales as the sister to all other angiosperms.^29^ However, it was not clear which was the most basal angiosperm. Recent releases of new genome sequences has greatly improved phylogenomic or phylotranscriptomic analysis for species tree reconstruction.^30^ A phylogenetic analysis of 61 plastid genes first reported Nymphaeales and the Amborella, the extant relatives, as the most basal lineage of flowering plants.^31^ This was later supported by two phylotranscriptomic analyses.^9,10^ In the last few years, phylogentists have attempted to resolve which angiosperm is the most basal.

#### Amborella as the most basal angiosperm

Unlike single gene-based phylogenetics, when using three mitochondrial genes, one chloroplast gene and one nuclear gene, an early phylogenetic analysis placed *Amborella*, and not water lilies, as the most basal angiosperm branch^9^ (Figure 2). This species tree topology is well supported by two recent phylotranscriptomic analyses^9,10^ using nuclear genes and one phylogenomic analysis using plastid and mitochondria genes.

#### Water lilies and Amborella as the basal sister to all other angiosperms

In other studies, *Amborella* and water lilies have been thought to form sister groups that both represent the first lineage to all other angiosperms. Relying on both nuclear and plastid genes, Xi and colleagues in 2014 placed *Amborella* and water lilies as sister groups using the coalescent-based phylogenetic method, and these sister groups serve as the most basal angiosperm clade^32^ (Figure 2).

#### Water lilies as the most basal angiosperms

There is still evidence to support water lilies as the most basal angiosperm. Relying on concatenation-based phylogenetic analysis of the whole chloroplast coding genes and using the transversion of the third position of the codon, researchers found that the water lily was the earliest branch of all extant angiosperms.^13^ A comparison of the female gametophyte and the embryo-nourishing tissue ploidy also suggested that Amborella was an exception in the ANITA group, which contained triploid endosperm and nine cells in the embryo sac and is thereby closest to monocots and eudicots^13,33^ (Figure 2). In addition, water lilies contain fewer stomatal modifications from the ancestral angiosperm stomata, whereas *Amborella* exhibited extensive modifications of stomata.^34^ In addition, the first known fossil flower of a water lily is from the early cretaceous period, approximately 125–115 million years ago.^35^ Another Jurassic fossil with flowers and other above-ground organs including the archaefructus is also placed within Nymphaeales.^6^

#### Phylogenetic signals hold the key for basal angiosperm phylogeny

A major concern in phylogenomics is the selection of the best phylogenetic signals, which are now generally regarded to be low/single-copy nuclear genes^36^ that should fulfill two important criteria: high neutrality and low saturation.^13^ For the selected genes, position 1 and position 2 codons lack synonymous mutation rates and suffer extremely low neutrality.^13^ Researchers found that position 3 transversion rates are suitable for both shallow and deep phylogenetic tree constructions.^13^ Based on this position 3 transversion, most single-gene-based trees placed the water lily as the most basal lineage of angiosperms.^13^ For species tree construction for angiosperms, we suggest the utilization of both protein sequences and nucleotide sequences as a more accurate method for land plant species tree construction.^10^ Most importantly, phylogenetic signals for species tree reconstruction should be genome-wide and contain a large number of signals but not rare genes or a limited number of signals.

#### Concatenation VS coalescent methods

In recent years, phylogenomics have relied on both the concatenation method and coalescent method.^14,32^ Although the coalescent method is theoretically sound to explain incomplete lineage sorting, both theories and applications show that concatenation could yield misleading results when highly conflicting gene trees exist, due to incomplete lineage sorting. These two methods have been under heated debate regarding the effects of tree estimation error in phylogenomics.^37–41^ Strong phylogenetic signals are still needed for more accurate species tree inference.^42^

Until recently, our understanding of plant phylogeny has largely depended on studies of plastid, mitochondrial, and ribosomal genes. However, recently, using large-scale comparisons of dozens of genes comprising thousands of DNA bases, phylogenomics have reshuffled most of our long-established trees of life, such as trees for eukaryotic life,^43^ bird life,^44^ fish life,^45^ and major nodes of eudicots of plants.^46^ The availability of the *Amborella* genome^21^ has shed light on basal angiosperm tree construction, but definite resolution of basal angiosperm phylogeny has not been resolved.

### Pantropical water lilies could serve as the model for studying basal angiosperms

**Figure 3:**
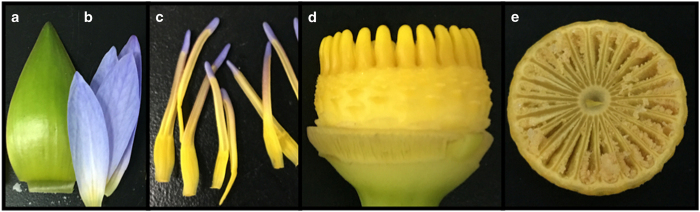
Floral organs of a typical tropical water lily. (**a**) petal, (**b**) sepal, (**c**) stamen, (**d**) carpels on the receptacle, (**e**) numerous young seeds.

To understand basal angiosperm evolution and the radiation of angiosperms, a good model species is needed. Among all basal angiosperms, the enormous genome size for Austrobaileyales, ~7050 Mb,^47^ is a major challenge for genome decoding and genetic experiments. A slow growth rate and the woodiness of Austrobaileyale plants and Amborella may also be challenging due to the difficulty of producing experimental materials. In the water lily order, *Cabomba* displays multiple features as a model for basal angiosperms, such as small size and rapid vegetative growth, but its large genome size, 3290 Mb,^47^ excludes it from gene functional studies, as large genomes usually harbor redundant gene copies, are highly heterogenetic and thereby not appropriate for gene functional studies. Although *Trithuria* species grow into small herbs, their genomes are still too large for genetic studies. Luckily, four pantropical diploid (2n=28) water lilies (or subgenus *Brachyceras*) may be good choices, as they have the smallest genomes, *N. caerulea=*567.24 Mb, *N. colorata=*489 Mb, *N. minuta=*449.88 Mb, *N. thermarum=*498.78 Mb.^17^ The native habitats of all four water lilies are in Africa, and all are annual plants (Table 2). *N. caerulea and N. colorata* are famous ornamental water lilies and have been widely used to breed new cultivars. *N. minuta and N. thermarum* are minute water lilies with thumb-sized flowers. Unlike hardy water lilies (a in Figure 1), these four tropical water lilies are easy to cultivate and maintain hundreds of plants in a single green house; it is easy to trigger flowering via temperature control (below 18 °C). All four water lilies can produce hundreds of seeds in a single flower (Figure 3) and can be used to generate a large mutant library. These plants are also easy to self-pollinate in nature to generate pure lines, and can also easily be cross-pollinated. They have a relatively short life cycle of approximately three months from seed to seed in tropical regions. In addition, *N. thermarum* has recently been well studied for its potential as a model system for basal angiosperms.^48^ These characteristics make these four water lilies the best candidates for genome sequencing and the best model for functional studies.

### The water lily genome for basic evolutionary research and applied horticulture

Based on the advantages of pantropical water lilies, we launched a genome-sequencing project of *N. colorata* using a third-generation single-molecule real-time sequencing method. We produced half of the reads >20 kb, and this has facilitated the assembly of complex repeating sequences and GC-rich regions that are usually highly fragmented or even unassembled in next-generation sequencing projects.^49^ We have annotated the genes and other key DNA elements using multiple tools. The future reference water lily genome will provide genomic information for reconstructing the karyotype of an angiosperm ancestor, with the species trees of basal angiosperms and early massive radiation of angiosperms. This availability of the water lily reference genome will greatly help us to understand Charles Darwin’s ‘abominable mystery’, the early evolution trajectory of angiosperms, the aquatic life style of angiosperms, the evolution of a 4-celled embryo sac and diploid endosperm, the comparative analyses of genes and other elements such as conserved non-coding elements and telomeres. The water lily genome is also needed to revisit the age of angiosperms and whether they evolved 0.1 billion-years ago^50^ or 0.2 billion years ago.^51^ The reference genome will also provide genetic information for breeders and geneticists. Currently, only seven aquatic plants have their genomes decoded, and only two aquatic ornamental plants, the water lily and the sacred lotus, have sequenced genomes (Table 3). The similar appearance of the water lily and the lotus does not actually indicate a tight relationship; the former is a basal angiosperm and the latter is a eudicot. Thus, the genome of the water lily will serve as a template to accelerate genomic studies of other aquatic ornamentals.

## Conclusions

Upgrading sequencing technologies and bioinformatics tools have provided high-resolution genomic details, showing great potential for understanding the large questions in biology (including Darwin’s famous abominable mystery), and are valuable resources for molecular breeding. Although the genetics and genomics of water lilies are incipient, four pantropical water lilies, especially *N. colorata*, show great potential as a model system to study basal angiosperms; their genomes will greatly enhance our current knowledge, including Charles Darwin’s abominable mystery, the early evolutionary trajectory of angiosperms, the aquatic life style of angiosperms, and molecular breeding.

## Figures and Tables

**Table 1:** Available molecular research on water lilies

	*Taxon name*	*Chromosome (*n*)*	*Genome size (Mb)*	*Genetic study*	*Transcriptome*
Nymphaeaceae	*Nymphaea alba* L.	42 [1]	1950 [6]	INO gene [8], ITS2+matK [9]	
	*Nymphaea amazonum* Mart. & Zucc.	9 [1], ~18 [2]	821.52 [2]	ITS2+matK [9]	
	*Nymphaea ampla* (Salisb.) DC.	14 [2]	772.62 [2]		
	*Nymphaea atrans* S. W. L. Jacobs	42 [1]	1408.32 [2]		
	*Nymphaea bissetii* hort.	42 [1]			
	*Nymphaea caerulea* Savigny	14 [1]		ITS2+matK [9]	
	*Nymphaea carpentariae* ‘Julia Leu’	~42 [2]	1447.44 [2]		
	*Nymphaea candida* C. Presl	56 [1]	1936.44 [2]		
	*Nymphaea capensis* Thunb.	14 [1]		trnH-psbA, rpoC1 [10]	
	*Nymphaea colorata* Peter	14 [2]	489 [2]	INO gene [8]	
	*Nymphaea conardii* Wiersema	9 [1]			
	*Nymphaea daubeniana* Hort. ex O. Thomas.	28 [1]			
	*Nymphaea dentatamagnifica* Bisset.	42 [1]			
	*Nymphaea gardneriana* Planch.	14 [1]			
	*Nymphaea gigantea* Hook.	112 [1]	2709.06 [2]		
	*Nymphaea heudelotii* Planch.	14 [1]			
	*Nymphaea immutabilis* S. W. L. Jacobs	42 [1]	1408.32 [2]		
	*Nymphaea jamesoniana* Planch.	14 [1]		ITS2+matK [9]	
	*Nymphaea japono-koreana* Nakai	56 [1]			
	*Nymphaea lasiophylla* Mart. & Zucc.	9 [1]			
	*Nymphaea lingulata* Wiersema	9 [1]			
	*Nymphaea lotus* L.	28 [1]	1779.96/1682.16 [2]	ITS2+matK [9], trnH-psbA, rpoC1 [10]	
	*Nymphaea mexicana* Zucc.	28 [1]	586.80 [2]		
	*Nymphaea micrantha* Guill. & Perr.	14 [2]	889.98 [2]	ITS2+matK [9]	
	*Nymphaea minuta*	14 [2]	449.88 [2]		
	*Nymphaea nouchali* Burm.	38 [1], 42 [2]	1193.16 [2]	ITS2+matK [9], trnH-psbA, rpoC1 [10]	
	*Nymphaea nouchali* var. caerulea (Savigny) Verdc.	14 [1]	567.24 [2]		
	*Nymphaea novogranatensis* Wiersema	14 [1]		ITS2+matK [9]	
	*Nymphaea odorata* Aiton	28 [1], 56 [2]	1574.58 [2]	ITS2+matK [9]	
	*Nymphaea oxypetala* Planch.	42 [1]		ITS2+matK [9]	
	*Nymphaea pubescens* Willd.	28 [2]	1975.56 [2]		
	*Nymphaea prolifera* Wiersema	9 [1]			
	*Nymphaea pubescens* Willd.	12 [1]		ITS2+matK [9]	
	*Nymphaea rubra* Roxb. ex Andrews	42 [1]		ITS2+matK [9]	
	*Nymphaea rudgeana* G. Mey.	21 [1]	792.18 [2]		
	*Nymphaea stellata* var. versicolor (Sims) Hook. & Thomson	28 [1]			
	*Nymphaea sturtevantii* J. N. Gerard	28 [1]			
	*Nymphaea tenerinervia* Casp.	10 [1]			
	*Nymphaea tetragona* Georgi	42 [1]		ITS2+matK [9]	
	*Nymphaea tetragona* subsp. Leibergii / Nymphaea leibergii	56 [1]			
	*Nymphaea thermarum* E. Fisch.	14 [1]	498.78 [2]		
	Nymphaea tuberosa / Nymphaea odorata subsp. Tuberosa	42 [1]			
	*Nymphaea violacea*	56 [2]	1770.18 [2]		
	*Euryale ferox*	29 [1]	870.42 [2]	ITS2+matK [9]	
	*Nuphar advena* (Aiton) W. T. Aiton	17 [1]	2709.06/2718.84 [2]	ITS2+matK [9]	y [11]
	*Nuphar lutea* (L.) Sm.	17 [1]	2875.32 [2]	ITS2+matK [9]	
	*Nuphar microphylla* (Pers.) Fernald	17 [1]		ITS2+matK [9]	
	N*uphar polysepalum* Engelm.	17 [1]	3080.70/3070.92 [2]	ITS2+matK [9]	
	*Nuphar pumila* (Timm) DC.	17 [1]		ITS2+matK [9]	
	*Nuphar variegata* Durand	17 [1]		ITS2+matK [9]	
	*Nuphar* × *spenneriana* Gaudin	17 [1]	2581.92 [2]		
	*Nuphar intermedia* Ledeb.	17 [1]			
	*Nuphar japonica* DC.	17 [1]	2699.28 [2]	ITS2+matK [9]	
	*Nuphar subintegerrima* Makino	17 [1]		ITS2+matK [9]	
	*Barclaya longifolia*	17 [1]		ITS2+matK [9]	
	*Victoria cruziana*	12 [1]	4009.80 [2]	ITS2+matK [9]	
	*Victoria yamasu*	12 [1]			
	*Victoria amazonica*	10 [1]	4557.48 [2]	ITS2+matK [9]	
	*Victoria* ‘Longwood Hybrid’	11 [2]	4303.20 [2]	ITS2+matK [9]	
Cabombaceae	*Cabomba caroliniana*	52 [2]	3471.9 [2]	ITS2+matK [9]	
	*Brasenia schreberi*	36 [3], 40 [1], [2]	1193.16 [2]	ITS2+matK [9]	
Hydatellaceae	*Trithuria submersa*	28 [1]	~2680 [7]	ITS2+matK [9]	y [12]
	*Trithuria inconspicua*	12 [1]		ITS2+matK [9]	
	*Trithuria konkanensis*	20 [4]			
	*Trithuria australis*	7 [5]		ITS2+matK [9]	

[1] http://ccdb.tau.ac.il/ [2] Pellicer *et al.*,^17^ [3] Diao *et al.*,^16^ [4] Gaikward *et al.*,^52^ [5] Iles *et al.*,^53^ [6] Vialette-Guiraud *et al.*,^47^ [7] Kynast *et al.*,^54^ [8] Yamada *et al.*,^18^ [9] Biswal *et al.*,^4^ [10] Chaveerach *et al.*,^22^ [11] http://sra.dnanexus.com [12] Marques *et al.*,^55^

**Table 2:** Characteristics of four pantropical water lilies

*Species*	*Genome size*	*Chromosome*	*Classification*	*Distribution*	*Flowers in diameter*
*Nymphaea caerulea*	567.24 Mb	2*n*=28	*Nymphaea, Brachyceras*	East Africa	10–15 cm
*Nymphaea colorata*	489 Mb	2*n*=28	*Nymphaea, Brachyceras*	tropical East Africa	11–14 cm
*Nymphaea minuta*	449.88 Mb	2*n*=28	*Nymphaea, Brachyceras*	Madagascar	2 cm
*Nymphaea thermarum*	498.78 Mb	2*n*=28	*Nymphaea, Brachyceras*	Rwanda, Africa	10–15 cm

The four listed genome sizes and chromosome counts have been reported.^17^

**Table 3:** Sequenced aquatic plants

*Scieitific name*	*Common name*	*Classification*	*Ornamental plant*	*Habitate*	*Genome size*
*Nymphaea* spp.	Water lilies	Basal angiosperms	Partially yes	Floating leaf type	~400 Mb–2.7 Gb
*Nelumbo nucifera*	Sacred lotus	Basal eudicot	Yes	Emerged plant	929 Mb
*Utricularia gibba*	Floating bladderwort	Eudicot, Lentibulariaceae	No	Floating plant	82 Mb
*Zostera marina*	Seagrass	Monocot, Zosteraceae	No	Submergent plant	202.3 Mb
*Spirodela polyrhiza*	Duckweed	Monocot, Araceae	No	Free-floating	158 Mb
*Zizania latifolia*	Manchurian wildrice	Monocot, Poaceae	No	Wetland plant	586 Mb
*Oryza sativa*	Rice	Monocot, Poaceae	No	Wetland plant	420 Mb

The genome of *Nymphaea colorata* was sequenced recently by our team. Other genomes have been sequenced and are publicly available.
